# Capsaicin Causes Vasorelaxation of Rat Aorta through Blocking of L-type Ca^2+^ Channels and Activation of CB_1_ Receptors

**DOI:** 10.3390/molecules25173957

**Published:** 2020-08-30

**Authors:** Felipa Andrade, Cinthia Rangel-Sandoval, Alejandrina Rodríguez-Hernández, Evelyn López-Dyck, Alejandro Elizalde, Adolfo Virgen-Ortiz, Edgar Bonales-Alatorre, Georgina Valencia-Cruz, Enrique Sánchez-Pastor

**Affiliations:** 1 National Technological Institute of Mexico/Technological Institute of Colima, Avenida Tecnológico No. 1, CP 28976 Villa de Álvarez, Colima, Mexico; felipa.andrade@colima.tecnm.mx; 2 University Center for Biomedical Research, University of Colima, Apdo. Postal No. 11, CP 28040 Colima, Colima, Mexico; cinthiarangel4@gmail.com (C.R.-S.); elizalde_alex@ucol.mx (A.E.); avirgen@ucol.mx (A.V.-O.); ebonales0@ucol.mx (E.B.-A.); gvalencia6@ucol.mx (G.V.-C.); 3 Faculty of Medicine, University of Colima, Apdo. Postal No. 11., CP 28040 Colima, Colima, Mexico; arodrig@ucol.mx; 4 State University of Sonora, Navojoa Academic Unit. Blvd. Manlio Fabio Beltrones 810, CP 85875 Navojoa, Sonora, Mexico; janydyck2000@hotmail.com

**Keywords:** cannabinoid receptor 1, TRPV1, Capsaicin, vasorelaxation

## Abstract

The aim of this work was to determine whether Capsaicin may exert a vascular regulation through the activation of CB_1_ and/or CB_2_ receptors causing vasorelaxation in the rat aorta. Our results show the location of TRPV1 mainly in the endothelial and smooth muscle cells membrane. Nevertheless, Capsaicin caused vasorelaxation of this artery through a mechanism independent of TRPV1, since the specific antagonists Capsazepine and SB-366791 did not block the effect of Capsaicin. Because the significant expression of CB_1_ and CB_2_ receptors has been previously reported in the rat aorta, we used antagonists for these two receptors prior to the addition of Capsaicin. In these experiments, we found that the inhibition of CB_1_ using AM281, decreases the vasorelaxant effect caused by Capsaicin. On the other hand, the vasorelaxant effect is not altered in the presence of the CB_2_ receptor antagonist AM630. Furthermore, a partial decrease of the effect of Capsaicin was also seen when L-type calcium channels are blocked. A complete block of Capsaicin-induced vasorelaxation was achieved using a combination of Verapamil and AM281. In accordance to our results, Capsaicin-induced vasorelaxation of the rat aorta is neither dependent of TRPV1 or CB_2_ receptors, but rather it is strongly suggested that a tandem mechanism between inactivation of L-type calcium channels and the direct activation of CB_1_ receptors is involved. These findings are supported by CB_1_ docking simulation which predicted a binding site on CB_1_ receptors for Capsaicin.

## 1. Introduction

Blood pressure mainly depends on vascular tone, which is regulated by several mechanisms. These mechanisms include the release of some substances from the inner lining of the arteries such as NO, which causes dilation of the arteries or endothelin, which causes vasoconstriction [1]. Some substances cause vasorelaxation by interacting with ion channels in the medial layer or with some receptors such as Vanilloid, CB_1_, CB_2_, and/or GPR55 receptors.

Vanilloid receptors are members of the Transient receptor potential channels (TRP channels) superfamily, which are non-selective cation channels and are found in different tissues, such as the skin, intestine, airways, and epithelial and endothelial cells [2]. TRPV1 is expressed in the smooth muscle and endothelium of the aorta and also in some other arteries, therefore, this receptor may have a very important role in the regulation of vascular tone [3]. These receptors can be activated by ligands such as capsaicinoids—one of these compounds is capsaicin (a component of hot peppers)—by endogenous ligands such as the endocannabinoid anandamide, and by other factors such as acidic pH (<5.3) and high temperatures (>43 °C) [2].

On the other hand, cannabinoid receptors (CB_1_ and/or CB_2_) are activated by endogenous cannabinoids (endocannabinoids) and exogenous cannabinoids, which are the active components of Cannabis sativa (marijuana). Both endocannabinoids and exogenous cannabinoids and synthetic cannabinoids cause vasorelaxation not only by interacting on cannabinoid receptors, but also by interacting with some other receptors (TRPV1, GPR55) or by their direct action on ion channels. Therefore, vanilloid and cannabinoid receptors may be related to the regulation of vascular tone, causing the vasorelaxation of some arteries, thus participating in the regulation of blood pressure [4,5,6].

The main TRPV1 agonist (Capsaicin) can cause vasorelaxation in several artery preparations as well. However, to date, the mechanisms of action of Capsaicin in modulating vascular tone is controversial. Although there are some reports implicating the role of TRPV1 in modulating vascular tone, some research has reported that Capsaicin causes vasorelaxation of rat aorta, rabbit coronary artery and human and porcine arteries by mechanisms independent of TRPV1 [5,7,8].

Therefore, the aim of the present work was to further investigate the different mechanisms involved in the effects of Capsaicin on the vascular tone of the rat aorta by determining the possible role of cannabinoid receptors in vasorelaxation caused by Capsaicin in the rat aorta. Here, we present evidence that Capsaicin induces vasorelaxation of the rat aorta by blocking L-type calcium channels and activating CB_1_, but not by activation of TRPV1 or CB_2_ receptors.

## 2. Results

### 2.1. Expression of TRPV1 Receptors in Smooth Muscle and Endothelial Cells of Rat Aorta

**Figure 1:**
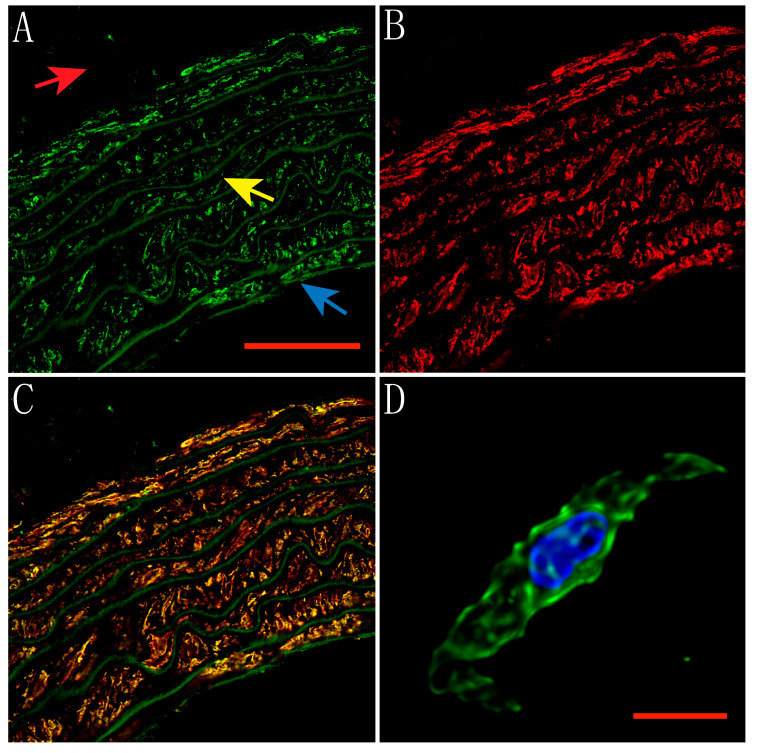
Expression of TRPV1 receptors in the smooth muscle of the rat aorta. (**A**) TRPV1 receptors labeled with FITC (Green). The scale bar corresponds to 50 μm. (**B**) Aortic ring labeled with anti-smooth muscle α-actin conjugated with Alexa Fluor 568 (Red). (**C**) Overlay showing the high co-localization (Yellow) of TRPV1 receptors in smooth muscle. The arrows indicate the arterial layers. The adventitia is marked with red, the tunica media with yellow and the tunica intima with blue. (**D**) Aortic smooth muscle cell labeled with anti-TRPV1 (Green) and stained with DAPI (Blue). The scale bar corresponds to 10 μm.

Capsaicin is a known TRPV1 receptor agonist. These receptors are expressed in several rat arteries [9], however, their location in the smooth muscle or endothelial cell membrane where they can be activated and play a role in regulating vascular tone is unclear. Therefore, immunostaining followed by confocal microscopy experiments was used to evaluate the location of TRPV1 receptors in rat aortic rings. Thus, here we show the expression of TRPV1 receptors in both smooth muscle cells and endothelial cells of the rat aorta. Figure 1 shows an aortic ring labeled with polyclonal rabbit anti-TRPV1 (Figure 1A, green) and anti-smooth muscle α-actin (Figure 1B, red). TRPV1 receptors are clearly located on smooth muscle cells as it can be seen as yellow color in the overlay (Figure 1C). A dispersed smooth muscle cell of the aorta is shown in Figure 1D, TRPV1 receptors (green) are expressed on the cell surface, as well as in the cytoplasm and nucleus (blue).

**Figure 2:**
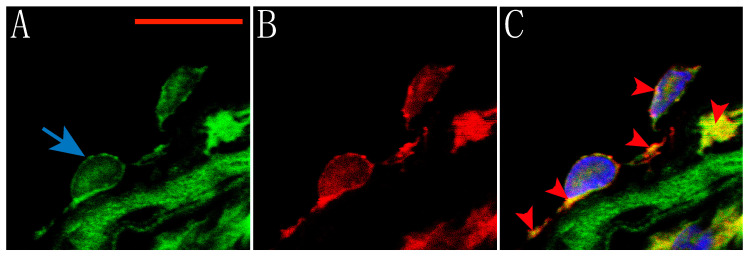
Expression of TRPV1 receptors in rat aorta endothelium. (**A**) TRPV1 was detected by a specific antibody and a FITC-conjugated secondary antibody (Green). The scale bar corresponds to 5 μm. (**B**). The tissue was co-labeled with an endothelium-specific antibody and a secondary antibody conjugated with Alexa Fluor 568; the cells are located along the inner layer, indicated by a blue arrow. (**C**) The superimposition of the images highlights the co-localization of the receptor in the endothelial cells (Arrowheads). Nuclei were stained using Prolong Gold antifade with DAPI.

Furthermore, co-labeling of the endothelial cell layer in an aortic ring with polyclonal rabbit anti-TRPV1 (green) and monoclonal mouse anti-endothelial cells (red) is shown on Figure 2. Thus, the location of these receptors on endothelial cells can be seen. Nuclei were stained using DAPI (Blue). Our results corroborate the expression of TRPV1 receptors in both smooth muscle and endothelial cells of the rat aorta and show evidence of their location in the membrane of these cells.

### 2.2. Capsaicin-Induced Vasorelaxation of the Rat Aorta Does not Involve TRPV1 Receptors and Is Partially Endothelium Dependent

**Figure 3:**
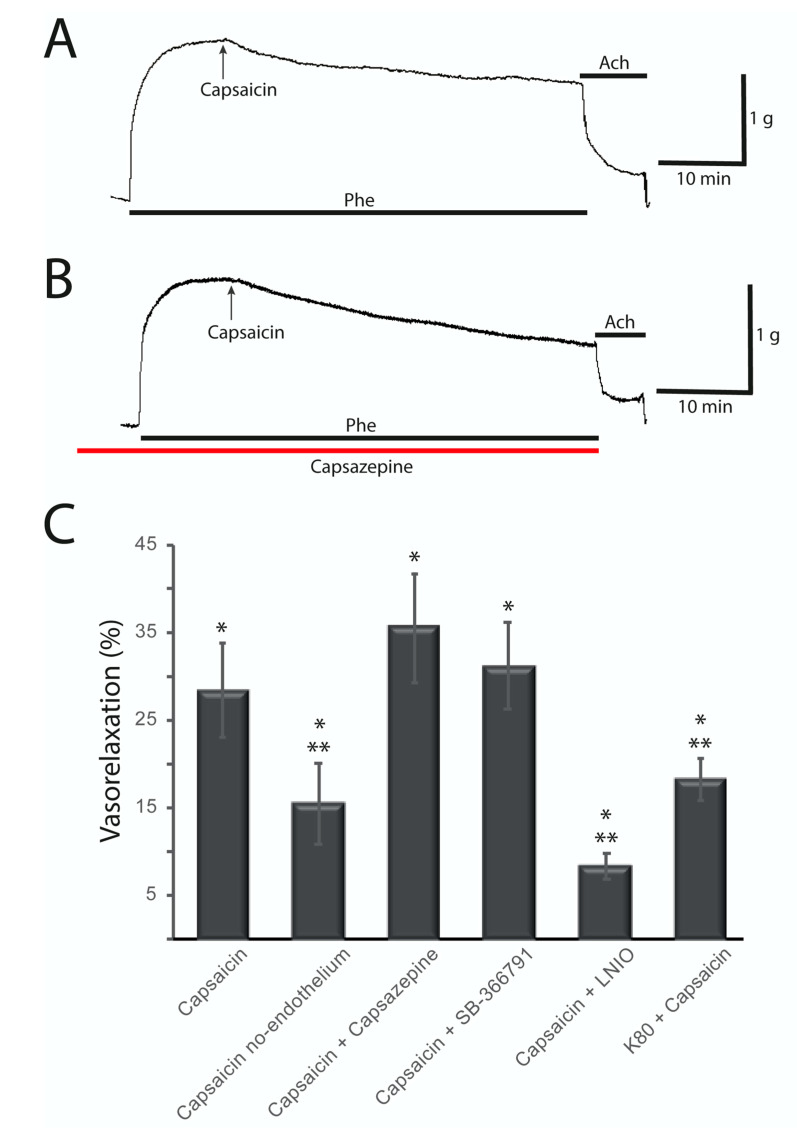
Vasorelaxant effect induced by Capsaicin 10 µM. (**A**) Typical recording showing the vasorelaxant response to Capsaicin in a pre-contracted rat aortic ring with endothelium. Black arrows indicate the addition of Capsaicin. The TRPV1 agonist Capsaicin 10 µM causes a vasorelaxant effect of 28.33 ± 5.41% (relative to Phe pre-contraction; *p* < 0.01; *n* = 20 rings, 10 rats). (**B**) Representative trace showing that Capsazepine 10 μM does not antagonize the vasorelaxant effect observed with Capsaicin (35.63 ± 6.32%; *p* < 0.01; *n* = 9 rings, 7 rats). The red line indicates the addition of Capsazepine. (**C**) Summary of the effect induced by Capsaicin under different experimental conditions. * indicates a significant change in tension compared to Phe pre-contraction. ** indicates a significant change compared to the effect of Capsaicin alone.

To determine the participation of vanilloid receptors in the regulation of the vascular tone of the rat aorta, the agonist capsaicin was used to activate these receptors. Experiments were performed on aortic rings with or without pre-constriction. Phenylephrine (Phe) 1 µM was used to pre-contract the aortic rings. Concentration-dependent vasorelaxation of rat aortic rings with Capsaicin was reported by Hopps et al. [5], with a pEC50 = 6.42 ± 0.14 and Torres-Narvaes et al. [10] studied the effect of Capsaicin 10 and 30 μM. Thus, in this work, the effect of Capsaicin 10 μM was tested. After 1 h in the presence of Capsaicin 10 μM there was a slight increase on basal tension of aortic rings without pre-constriction of 0.02 ± 0.01 g (1% of change) which was no significant (*n* = 12 rings from six rats). On the other hand, Capsaicin 10 μM elicited a vasorelaxant response in intact aortic rings pre-contracted by Phe. Figure 3A shows a typical record of the vasorelaxant response to Capsaicin in a pre-contracted rat aortic ring with endothelium. This vasorelaxant effect was 28.33 ± 5.41% (Figure 3C), relative to the Phe contracture plateau (*p* < 0.01; *n* = 20 rings from ten rats). The red arrows indicate the addition of Capsaicin and the dark line indicates the time of exposure to Phe. Moreover, the vasorelaxant effect of Capsaicin was partially reversed on endothelium-denuded aortic rings (Figure 3C; 15.58 ± 4.64%; *p* = 0.001; *n* = 14 rings from seven rats). Therefore, Capsaicin displays a lower vasorelaxant effect on aortic rings without endothelium.

In order to determine the role of TRPV1 receptors in the vasorelaxant effect caused by Capsaicin, 30 min before Phe pre-contracture, the rings were incubated with the antagonist Capsazepine. Capsazepine (1 or 10 μM) does not antagonize the vasorelaxant effect observed with Capsaicin as shown in the representative trace in Figure 3B, indicating that its effect is not mediated by TRPV1 receptors (35.63 ± 6.32%; *p* < 0.01; *n* = 9 rings from seven rats). The red line indicates exposure to Capsazepine, while the dark line indicates exposure to Phe and the black arrow indicates the addition of Capsaicin. Further experiments were performed using a more potent antagonist, SB-366791 1 μM (Figure 3C), and no significant change on the effect of Capsaicin was observed (31.20 ± 4.96%; *p* < 0.01; *n* = 12 rings from six rats), corroborating that TRPV1 receptors are not involved on the vasorelaxant effect observed in our experiments. Thus, TRPV1 receptors do not have a role in the vasorelaxant effect induced by Capsaicin. Nevertheless, more experiments are needed in order to evaluate the role of these receptors in these arteries as they are highly expressed in the membrane of smooth and endothelial cells.

Additionally, it has been previously shown that Capsaicin can induce the release of Nitric oxide in the endothelial cells of the arteries, for which reason an inhibitor (L-NIO) of nitric oxide synthase was used. After incubating with L-NIO 100 μM, Capsaicin induces a lower vasorelaxant effect (Figure 3C), suggesting that a machinery of NO release is involved in the effect induced by Capsaicin.

Furthermore, to see the possible involvement of K^+^ channels in the Capsaicin-induced relaxation mechanism, we conducted experiments inducing aortic rings contractures by using a high potassium depolarizing external solution instead of Phe. Under these conditions, Capsaicin 10 μM has a smaller vasorelaxant effect reaching 18.28 ± 2.41% (Figure 3C; *p* < 0.01; *n* = 10 rings, five rats), pointing out a role for K^+^ channels in the mechanism of vasorelaxation induced by Capsaicin.

### 2.3. Capsaicin-Induced Rat Aortic Ring Vasorelaxation Involves CB_1_ Receptors and Voltage-Gated Calcium Channels

**Figure 4:**
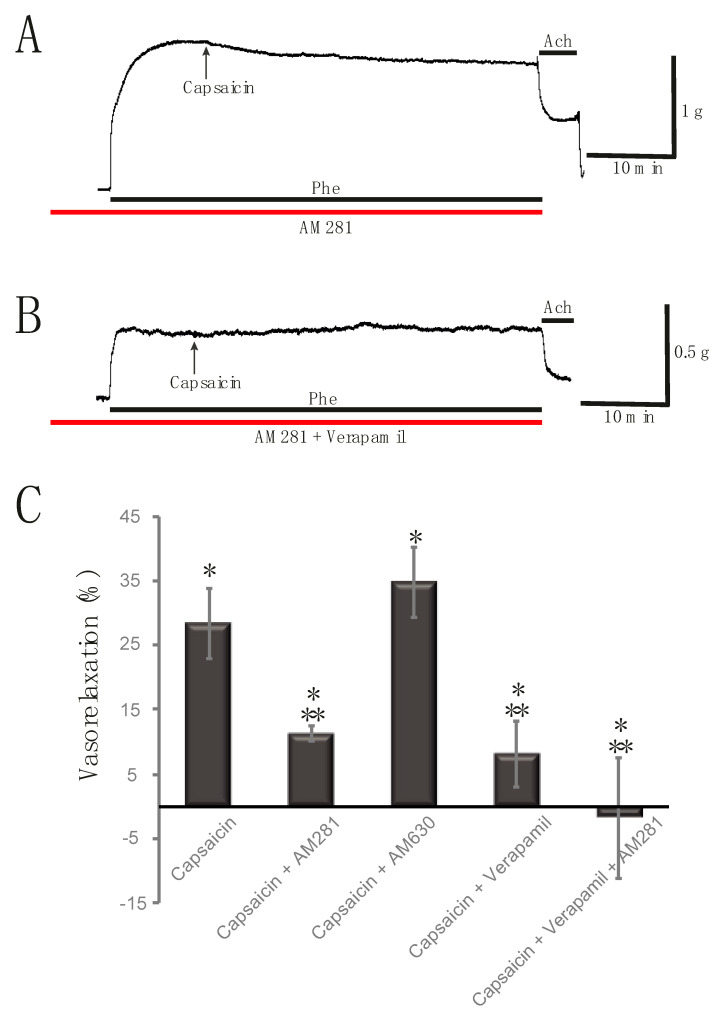
Effect of antagonists on Capsaicin-induced vasorelaxation. (**A**) Typical record showing that AM281 5 μM partially antagonizes the Capsaicin-induced vasorelaxant effect (11.32 ± 1.24%; *p* < 0.01; *n* = 20 rings, 9 rats). (**B**) Representative trace showing that Verapamil and AM281 together prevent the vasorelaxant effect of Capsaicin causing slight vasoconstriction (1.80 ± 9.38%; *p* = 0.85; *n* = 13 rings, 7 rats). The black line shows the exposure to Phe. Black arrows indicate the addition of Capsaicin. Red lines indicate exposure to each of the antagonists. (**C**) Summary of the action of antagonists on the vasorelaxant effect of Capsaicin. * indicates a significant change in tension compared to Phe pre-contraction. ** highlights a significant change compared to the effect of Capsaicin alone.

To further elucidate the mechanisms of action of Capsaicin in the vasorelaxation of the aortic rings, the tissues were previously incubated using CB_1_ or CB_2_ receptors antagonists. When a selective CB_1_ receptor antagonist AM281 5 μM is used, it partially antagonizes the vasorelaxant effect induced by Capsaicin, indicating that its effect involves the activation of CB_1_ receptors (11.32 ± 1.24%; *p* < 0.01; *n* = 20 rings from nine rats). A representative record showing the effect of Capsaicin in the presence of AM281 is shown in Figure 4A. The black arrow indicates the addition of Capsaicin, while the red line indicates the exposure to AM281, and the dark line indicates the presence of Phe. On the other hand, when using AM630, a CB_2_ antagonist, Capsaicin evokes a similar effect causing vasorelaxation of the aortic rings (Figure 4C; 34.74 ± 5.48%; *p* < 0.01; *n* = 12 rings from seven rats), indicating that CB_2_ receptors are not being activated by Capsaicin. It is known that AM281 may act as a CB_1_ inverse agonist [11], thus control experiments using AM281 alone were performed and no effect on the pre-contractured aortic rings was observed.

To further characterize the effect of Capsaicin, experiments were performed by blocking L-type calcium channels with a selective antagonist. The aortic rings were previously incubated with Verapamil 10 μM prior to induction of Phe pre-contracture. After blocking these calcium channels, Capsaicin 10 μM has a lower vasorelaxant effect on the tension of the aortic rings (Figure 4C; 8.13 ± 5.05%; *p* = 0.11; *n* = 9 rings, five rats), exhibiting a role of these calcium channels in the mechanism of the Capsaicin-induced vasorelaxation response. Additional experiments were performed by incubating aortic rings with both AM281 and Verapamil. Under these conditions, the vasorelaxant effect of Capsaicin 10 μM was completely blocked, which still resulted in a slight vasoconstriction of 1.80 ± 9.38% (*p* = 0.85; *n* = 11 rings, six rats). Therefore, blocking of L-type calcium channels and CB_1_ activation are key steps for Capsaicin-induced vasorelaxation of the rat aorta. A representative trace from this experimental series is shown in Figure 4B. A summary of the vasorelaxant effect of Capsaicin in the presence of antagonists is shown on Figure 4C.

### 2.4. Capsaicin and AM281 Binding Site Regions in the Human CB_1_ Receptor Interpose at the Same Binding Cavity

**Figure 5:**
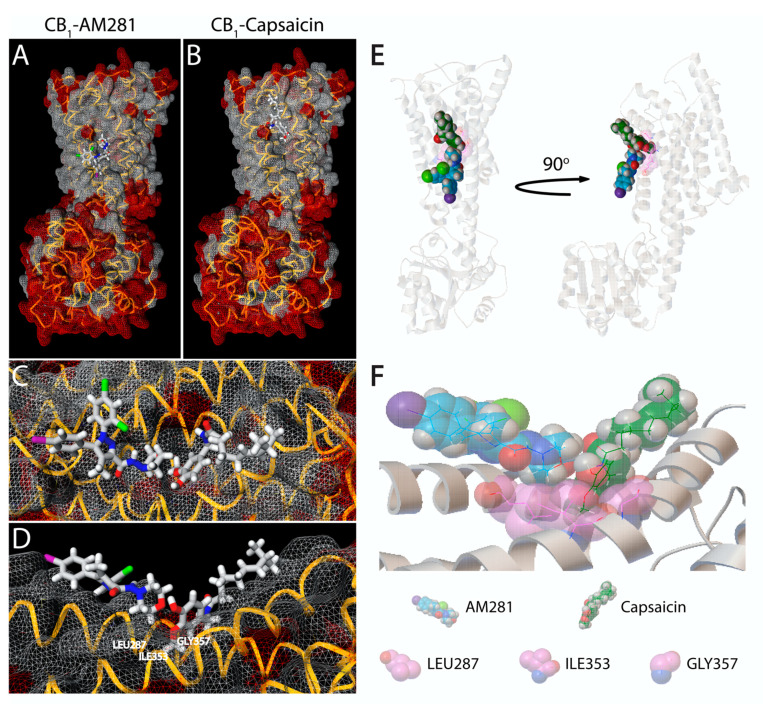
AM281 and Capsaicin binds to human CB_1_ receptor at the same binding region. (**A**,**B**) CB_1_ docking simulation presenting the predicted binding of AM281 and Capsaicin, respectively. (**C**) Front view and (**D**) Side view of CB_1_-AM281 and CB_1_-Capsaicin binding regions. (**E**) Superimposition of the two ligands models showing CB_1_ receptor displaying ribbon structure and the ligand molecules displaying atomic spheres (AM281 in blue and Capsaicin in green), as well as the cavity formed by the relevant residues in pink. (**F**) Close up of the two-model superimposition showing that the methoxy moiety of Capsaicin is internalized in the cavity formed by LEU287, ILE353 and GLY357.

Some endocannabinoids and phytocannabinoids act not only on CB_1_ or CB_2_ receptors, but also on TRPV1. Such is the case of Cannabidiol (CBD), which has a similar potency than Capsaicin for human TRPV1 [12] and Anandamide (AEA) which was the first endogenous agonist of TRPV1 showing a similar binding affinity [13,14] and the same binding site than Capsaicin [15]. These facts, together with the combination of the results of the present study, open the question of whether Capsaicin could act directly on cannabinoid receptors. To support this assumption; we performed simulations of molecular dynamics in silico using the inactive state crystalized structure of the human CB_1_ receptor (PDB ID 5U09) to compare and analyze the binding position of AM281 and Capsaicin at CB_1_ receptors. Interestingly, both molecules bind to CB_1_ and predicts slightly higher affinity for AM281 (best fitting score of −6.2) than the predicted for Capsaicin (−5.0; Figure 5A,B). Surprisingly, the binding poses of both components interpose in the same region of the cavity formed by the molecular topology of residues LEU287, ILE353 and GLY357 (Cav1) as revealed in the superimposition of the two docking simulations (Figure 5C (Front view) and D (Side view)). Taking into account that AM281 and Capsaicin could be present in the same binding region simultaneously (Figure 5E), Capsaicin would compulsorily need to fill Cav1 with its methoxy moiety in order to get the correct binding pose (Figure 5F), which would be improbable to occur once AM281 has docked into its own binding pose.

## 3. Discussion

TRPV1 vanilloid receptors play a key role in regulating Ca^2+^ permeability [16] and can be activated by Capsaicin, some endogen ligands such as Anandamide (which also activates CB_1_ receptors) and by mediators of inflammation [2]. Activation of the vanilloid receptors causes an intracellular increase in Ca^2+^ that results in the release of neuropeptides such as Calcitonin gene-related peptide (CGRP) and substance *p*. These neuropeptides are capable of regulating vascular smooth muscle since CGRP causes hyperpolarization of smooth muscle by activating K^+^ channels [5,17].

Capsaicin is well known to cause vasorelaxation in several types of blood vessels [5,18,19,20]. Since Capsaicin is a known TRPV1 agonist, its mechanism of action has been postulated to involve activation of these receptors to induce vasodilation. Several studies have recognized the participation of TRPV1 receptors in the regulation of vascular tone [3,10,13,21,22,23,24].

Although there are reports implicating the role of TRPV1 in modulating vascular tone, some studies have found that Capsaicin causes vasorelaxation by a mechanism independent of TRPV1 [5,7,8]. Capsaicin administration releases neuropeptides from sensory nerves, which have an important role in regulating vascular tone. In addition, Capsaicin shows different effects on contractility and ion channel activity in smooth muscle depending on the model and tissue preparation studied. Lo et al. (1995) [25] found that Capsaicin blocks L-type Ca^2+^ channels and suggested that it is the main mechanism of the observed vasorelaxation. In 2001, Yeon et al. [7] demonstrated that Capsaicin relaxes bronchial smooth muscle through Ca^2+^ channel blockade and K^+^ channels activation causing constriction in the cerebral arteries due to increased intracellular Ca^2+^. Some other studies have also shown that Capsaicin can affect vascular tone by different mechanisms besides activation of TRPV1 receptors. Gupta et al. (2007) [8] conducted experiments with isolated human and porcine arteries and found that TRPV1 receptors and the neuropeptide CGRP are not involved in the vasorelaxant effect produced by Capsaicin, since the competitive antagonist Capsazepine did not block its effect and was not affected by Olcegepant either, a selective CGRP antagonist. This implies that the release of neuropeptides does not play a role in the vasorelaxant effect of Capsaicin either.

In our conception, we began to carry out experiments with the natural TRPV1 receptor agonist Capsaicin, which caused vasorelaxation of the aortic rings in our experimental conditions. The effect observed using Capsaicin 10 μM was similar to the reported previously [5]. However, when we performed experiments blocking TRPV1 receptors using two specific antagonists (Capsazepine and SB-366791), the vasorelaxant effect observed with Capsaicin was not blocked; conversely, this effect was even greater in the presence of Capsazepine, which could be due to the possibility that Capsazepine may be blocking the voltage-gated Ca^2+^ channels causing greater vasorelaxation [26] or the fact that calcium influx through TRPV1 stimulates eNOS activity. So, the block by Capsazepine could cause an imbalance in the vasoconstriction–vasodilation status slightly towards relaxation [27]. In this way, these results demonstrate that the vasorelaxant effect in this case is not produced by the activation of TRPV1 receptors suggesting an unreported mechanism of Capsaicin to induce vasodilatation.

Our results also show a partial dependence on the endothelium (Figure 3C). It has been suggested that Capsaicin may induce vasorelaxation of the aorta by inducing NO release [10,28,29]. In order to probe it, an inhibitor of Nitric oxide synthase was used showing that inhibition of NOS by L-NIO partially blocked the vasorelaxant effect of Capsaicin, indicating the role of NO release in the vasorelaxation mechanism caused by Capsaicin.

Moreover, TRPV1 receptors and cannabinoid receptors share some signaling pathways in various cell types [30,31,32,33,34,35]. Several groups have demonstrated the activation of TRPV1 receptors by endocannabinoids, so there is a possibility that Capsaicin may activate cannabinoid receptors as well [32,36]. In this way, the effect of Capsaicin was explored after inhibiting CB_1_ or CB_2_ receptors with specific antagonists. Inhibition of the CB_2_ receptors caused no changes in the vasorelaxant effect of Capsaicin, suggesting these receptors have no role in the Capsaicin-induced vasorelaxation mechanism proposed. However, by blocking the action of CB_1_ receptors, the effect of Capsaicin was decreased for more than half, so it can be pointed out that the studied effect does depend on the activation of these receptors. Additional analysis was performed to determine the possible site of interaction of Capsaicin on CB_1_ receptors in order to support these findings. Combining the CB_1_ 3D structure and modeling data together with the pharmacology results, we speculate that Capsaicin could only exert an effect on CB_1_ if AM281 is not present. This is supported by the predicted higher binding affinity and the projected orientation pose that shows an interposition in a considerable segment of the Cav1 (LEU287, ILE353 and GLY357) region. Although the analysis was done using the sequence of Human CB_1_ receptor, it was corroborated that this region is also present on the Rat CB_1_ receptor sequence. This region, according to the prediction, is essential for the Capsaicin complete access to its binding site.

To further investigate the mechanism of action of the vasorelaxant effect of Capsaicin on rat aorta, experiments were performed by blocking ion channels which are modulated after activation of CB_1_ receptors. We have previously shown that activation of CB_1_ receptors located in the smooth muscle of the aorta and mesenteric arteries leads to activation of BK_Ca_^2+^ channels and blockade of L-type calcium channels [37,38]. The role of activation of K^+^ channels was evaluated by increasing the extracellular concentration of potassium thereby causing a depolarization. Under these conditions, the high potassium solution disrupts the potassium gradient through the membrane, decreasing the participation of potassium channels. Capsaicin has a lower vasorelaxant effect in high potassium contractures, pointing out that activation of K^+^ channels is a component of the mechanism to induce vasorelaxation by Capsaicin. Furthermore, blocking L-type calcium channels with Verapamil partially decreased the effect of Capsaicin as well, which is consistent with previous reports [5,25]. The effect was completely reversed by blocking these channels and CB_1_ receptors together. Therefore, the vasorelaxant effect of Capsaicin is triggered by its direct interaction with the L-type calcium channels located in the membrane of smooth muscle cells of the aorta as previously reported [5] and by the interaction with CB_1_ receptors causing its activation. These events bring on the following steps in the signaling mechanism that involve modulation in the release of NO, the activation of K^+^ channels (probably BK_Ca_^2+^ channels) and further inactivation of L-type calcium channels, leading to vasorelaxation of the aorta.

In conclusion, our results show that, besides inactivation of L-type calcium channels, Capsaicin also activates CB_1_ receptors causing vasorelaxation of the aorta, without involving TRPV1 receptors.

## 4. Materials and Methods

### 4.1. Animals

Adults male Wistar rats were used, which were kept in 12-h light cycles with food and water ad libitum. All procedures were performed following the Guide for the Use of Laboratory Animals (1996) and NOM1005 for bioethics and care of experimental animals.

### 4.2. Dissection

Rats were anesthetized by intraperitoneal administration of sodic pentobarbital in a single dose of 45 mg/kg and placed on a dissection table in a dorsal decubital position. The thoracic aorta was carefully dissected and placed in a petri dish containing a cold Krebs–Henseleit (KHS) solution bubbled with a mixture of 95% of O_2_ and 5% of CO_2_ and cleaned by using dissection material under the microscope.

### 4.3. Experimental Procedure

The thoracic aorta was cut into rings 2 to 3 mm long. The aortic rings were placed between two stainless steel wires, one of them held in a fixed support and the other hanging from a force transducer (Radnoti, Monrovia, CA, USA). For all the experiments, the rings were kept on KH solution at 37 °C, bubbled with a mixture of 95% of O_2_ and 5% of CO_2_ and stabilized at 2 g of basal tension. At the beginning of the experiments, a high potassium contracture (80 mM) was induced to verify the good condition of the tissue. To assess the effect of Capsaicin on the aortic rings, a pre-contraction was induced with Phenylephrine (1 μM) and once the contracture reached the plateau, Capsaicin was added to the tissue bath to monitor its effect for 1 h. An experimental set including four force transducers was used to perform experiments in parallel by duplicate. The force transducers were connected to a Digidata (Molecular Devices, Sunnyvale, CA, USA) through two Cyberamp 320 amplifiers (Molecular Devices). Data were acquired using the pClamp 9 Axoscope subroutine (Molecular Devices) and analyzed using the Clampfit subroutine. Data were expressed as the mean ± standard error. The sample size was 8 to 20 rings from at least 4 different rats.

### 4.4. Immunolabeling and Confocal Microscopy

Dissection of the aorta was performed as previously described. The complete thoracic aorta was fixed with 4% Paraformaldehyde (PFA) at 4 °C for 24 h. The tissue was then embedded in paraffin, cut into 3- or 10-mm rings, and placed on glass slides. To remove the paraffin, the rings were washed 3 times for 5 min with Xylene (Sigma) followed by 3 washes for 5 min each with different dilutions of Ethanol (95%, 70%, 50%, and 30%). The PBS solution added with 10% Normal goat serum (NGS) and 0.2% Triton-X was used to block the tissues for 30 min. After blocking, the rings were incubated overnight with primary antibody against TRPV1 (1:100; Alomone, Jerusalem, Israel) diluted in PBS solution added with 1% NGS and 0.2% Triton-X. Anti-alpha smooth muscle actin (1:100; Abcam, Cambridge, MA, USA) or anti-endothelial cells [RECA-1] (1:100; Abcam) antibodies were also used. The primary antibodies were then removed by washing with PBS solution containing 1% NGS and 0.2% Triton-X and the samples were incubated with secondary antibodies goat anti-rabbit IgG H&L (FITC; 1:100; Abcam) and goat anti-mouse IgG (H + L) secondary antibody, Alexa Fluor 568 conjugate (1:50; Invitrogen, Carlsbad, CA, USA) for 2 h at room temperature in the dark. Finally, the samples were washed and mounted using ProLong Gold antifade on a coverslip (Molecular Probes, Eugene, OR, USA). Confocal images were taken using a Zeiss LSM700 Confocal Microscope using a Plan-Neofluar 40X/1.3 objective with a resolution of 0.078 μm/pixel. Four immunoassays were performed using aortas from different rats, and three images of each sample were obtained. Images were 3D blind deconvolved using AutoQuant X3 and processed using ZEN 2009 and Image J. The maximum projection made with several planes is shown in the Figures.

For aortic myocytes, the aorta was collected and cut into small pieces and exposed to enzymatic treatment with Papain (40 U/mL, Dithiothreitol (DTT) 1 mM, in Tyrode solution with BSA 2 mg/mL) for 1 h on ice, then placed for 15 min in a 37 °C water bath. After that, the tissue was rinsed and transferred to a Collagenase II solution and incubated for another 15 min at 37 °C. Cells were mechanically dispersed by pumping with a polished Pasteur pipette and plated on coverslips to proceed with fixation with 4% PFA for 20 min and immunostaining as mentioned above.

### 4.5. Solutions

KHS with the following composition was used to maintain viability of tissues in all experiments. NaCl 118 mM, KCl 5 mM, NaH_2_CO_3_ 25 mM, MgSO_4_ 12 mM, KH_2_PO_4_ 12 mM, CaCl_2_ 20 mM, glucose 2 g, adjusted to pH 7.4. Tyrode solution was composed of: NaCl 130 mM, KCl 5.4 mM, NaH_2_PO_4_ 0.6 mM, MgCl_2_ 1 mM, HEPES 10 mM, Glucose 5 mM and Taurine 20 mM, adjusted to pH 7.4. Capsaicin and Capsazepine were prepared as 10 mM stock solutions in ethanol. SB-366791 was dissolved in DMSO as 10 mM stock solution. L-NIO and Verapamil were dissolved in water at a concentration of 10 mM.

### 4.6. Structure-Based Simulation of CB_1_ Receptor Ligands

A 3D crystal structure PDB file for Rat CB_1_ receptor protein is not currently available. For choosing an adequate PDB file, bioinformatic analysis of similarity using BLASTP suite was performed to compare the rat CB_1_ AA sequence (Uniprot Entry: P20272) against PDB database at https://blast.ncbi.nlm.nih.gov/Blast.cgi. The human CB_1_ receptor (Uniprot Entry: P21554; PDB ID 5U09) was the best fit with 97.25% of similarity and the PDB file was downloaded from www.rcsb.org. Ligand SDF files were obtained from PubChem collection at https://pubchem.ncbi.nlm.nih.gov/. Binding poses of ligands in CB_1_ receptor were generated using the Molecular Docking tools of the MCule platform, utilizing the Vina docking algorithm developed by Trott and Olson [39]. The molecular docking simulations were also checked using AutoDock 4.2.6 and were also used to generate the superimposition of AM281-CB_1_ and Capsaicin-CB_1_ binding poses [40].

### 4.7. Statistics

The data are shown as the average of *n* experiments and were compared using Student’s *t*-test considering a *p* value < 0.05 as statistically significant.
